# Hydrocele of the Canal of Nuck Mimicking Inguinal Hernia in an Adult Woman: Ultrasound and Magnetic Resonance Imaging (MRI) Correlation

**DOI:** 10.7759/cureus.116648

**Published:** 2026-09-21

**Authors:** Praveen K Tamminana

**Affiliations:** 1 Department of Radiology, Gouri Devi Institute of Medical Sciences and Hospital, Durgapur, IND

**Keywords:** canal of nuck, female inguinal swelling, hydrocele, inguinal canal cyst, inguinal hernia mimic, magnetic resonance imaging, ultrasound

## Abstract

A 29-year-old woman presented with right-sided inguinal swelling associated with intermittent pain for 1.5 years. Ultrasound examination demonstrated tubular anechoic cystic lesions within the right inguinal canal extending toward the labium majus, with minimal posterior acoustic enhancement, partial compressibility, and no internal vascularity on Doppler imaging. Magnetic resonance imaging (MRI) revealed an elongated cystic lesion measuring approximately 6.3×2.2 cm, extending from the deep inguinal ring through the superficial inguinal ring to the right labium majus. The lesion was hyperintense on T2-weighted and short tau inversion recovery (STIR) sequences and hypointense on T1-weighted images, with thin internal septations and no definite communication with the peritoneal cavity. Based on the clinical and characteristic multimodality imaging findings, a diagnosis of hydrocele of the canal of Nuck was made. This case demonstrates the imaging features of this uncommon cause of female inguinal swelling and its differentiation from an inguinal hernia.

## Introduction

Hydrocele of the canal of Nuck is a rare condition in women resulting from the persistence of the processus vaginalis, an embryological extension of the parietal peritoneum that accompanies the round ligament through the inguinal canal into the labia majora [1]. Normally, this canal obliterates within the first year of life. Failure of obliteration may lead to the formation of a fluid-filled cystic structure, commonly referred to as hydrocele of the canal of Nuck.

Clinically, this entity typically presents as a painless or intermittently painful inguinal swelling and is frequently misdiagnosed as an inguinal hernia due to overlapping clinical features. Because of its rarity in adult women, it often poses a diagnostic challenge. Imaging plays a pivotal role in distinguishing this condition from inguinal hernia, lymphadenopathy, cystic lymphangioma, abscess, or other cystic lesions of the inguinal canal [2].

We report the case of a 29-year-old woman presenting with chronic right-sided inguinal swelling in whom ultrasound and magnetic resonance imaging (MRI) established the diagnosis of hydrocele of the canal of Nuck, highlighting the importance of radiological correlation in accurate preoperative diagnosis.

## Case presentation

A 29-year-old woman presented with a history of right-sided inguinal swelling associated with intermittent pain for approximately 1.5 years. The swelling was gradually progressive and was not associated with fever, trauma, or prior surgery. There was no history of bowel obstruction symptoms. Clinical examination raised suspicion of a right inguinal hernia.

Ultrasound examination of the right inguinal region revealed two well-defined tubular anechoic cystic lesions within the inguinal canal. The larger lesion, measuring 2.60×0.77 cm, was located at the level of the superficial inguinal ring and extended inferiorly toward the right labium majus (Figure 1). Another similar tubular cystic lesion, measuring 1.63×0.95 cm, was identified near the deep inguinal ring (Figure 2). The lesions demonstrated minimal posterior acoustic enhancement, partial compressibility, and no internal vascularity on color Doppler imaging. No bowel loops or omental fat were visualized within the inguinal canal.

**Figure 1:**
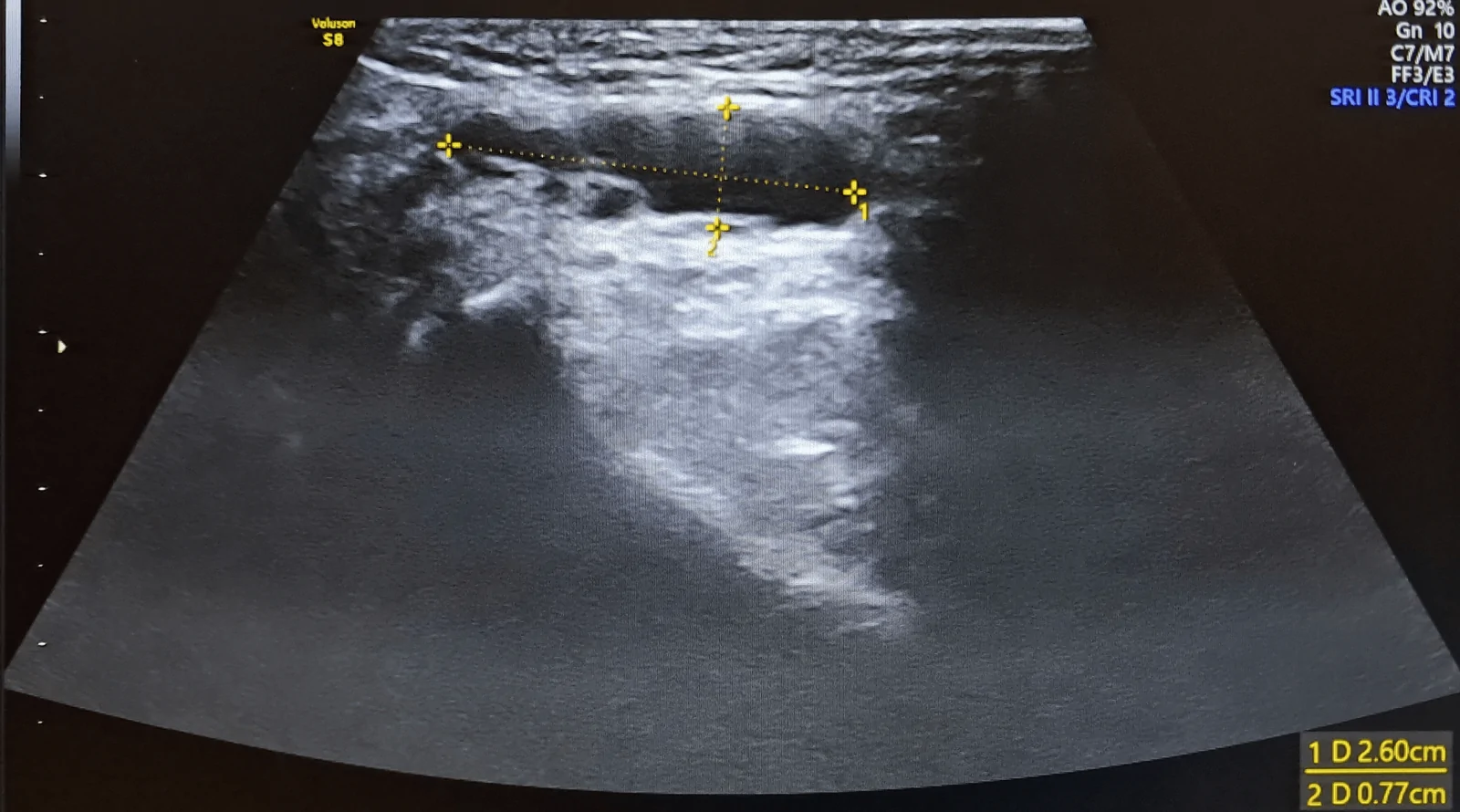
Ultrasound image of the right inguinal region at the level of the superficial inguinal ring Grayscale ultrasonography demonstrates a larger tubular anechoic cystic lesion measuring approximately 2.60×0.77 cm within the right inguinal canal. No bowel loops or omental fat are visualized within the inguinal canal. The lesion demonstrates mild posterior acoustic enhancement.

**Figure 2:**
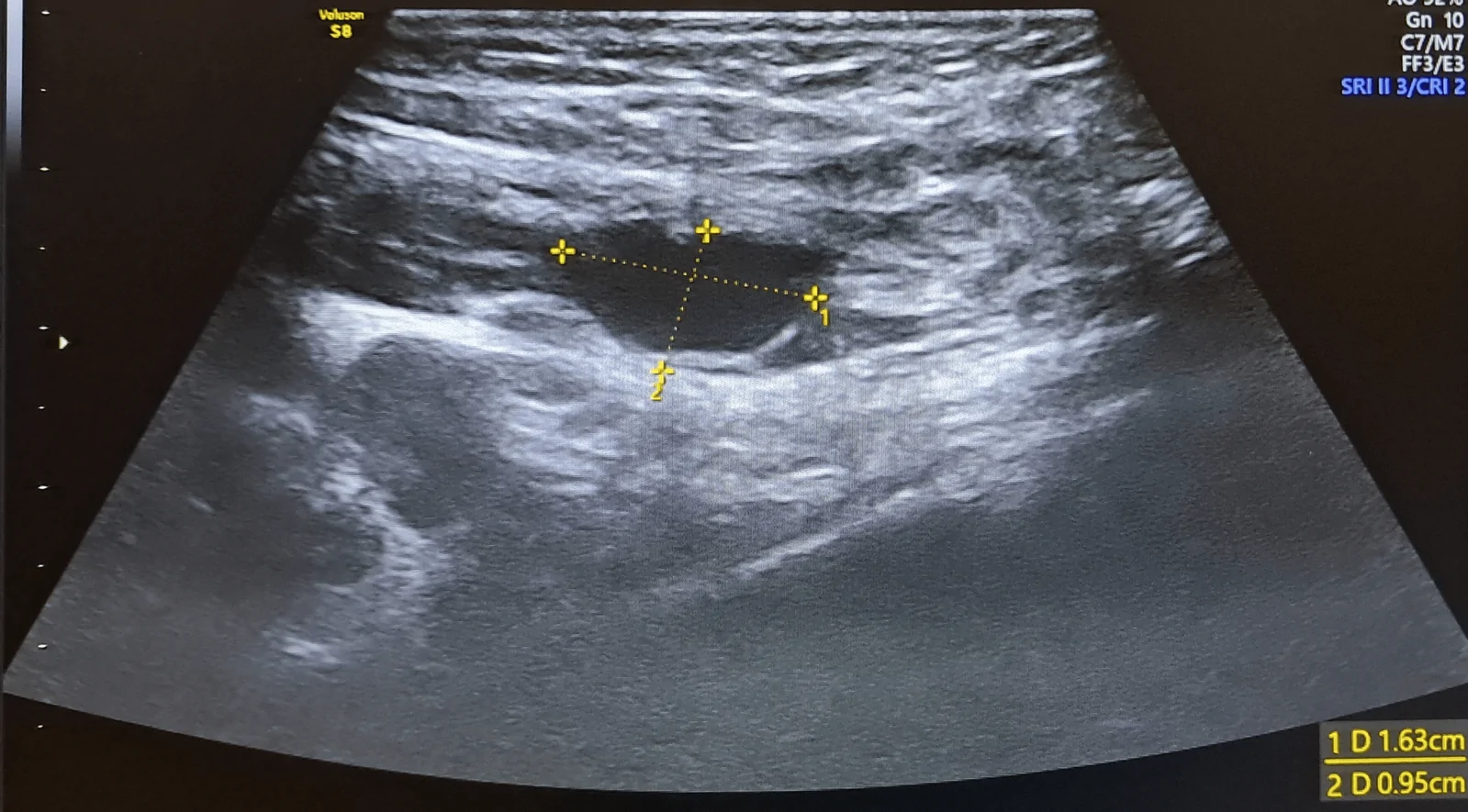
Ultrasound image of the right inguinal region at the level of the deep inguinal ring Grayscale ultrasonography shows a well-defined elongated tubular anechoic cystic lesion within the right inguinal canal measuring approximately 1.63×0.95 cm. The lesion shows thin walls and posterior acoustic enhancement without internal solid components.

Subsequent MRI of the pelvis demonstrated a tubular elongated cystic lesion within the right inguinal canal measuring approximately 6.3×2.2 cm in its entirety (Figure 3). The lesion extended from the region of the deep inguinal ring through the superficial inguinal ring up to the right labium majus. It appeared heterogeneously hyperintense on T2-weighted and short tau inversion recovery (STIR) sequences and hypointense on T1-weighted images, with a few thin internal septation-like areas (Figure 4). No definite communication with the peritoneal cavity was identified. No bowel loops, omental fat, or solid components were identified (Figure 5). The two cystic components seen on ultrasound were subsequently demonstrated on MRI to represent portions of a single elongated cystic lesion extending along the right inguinal canal.

**Figure 3:**
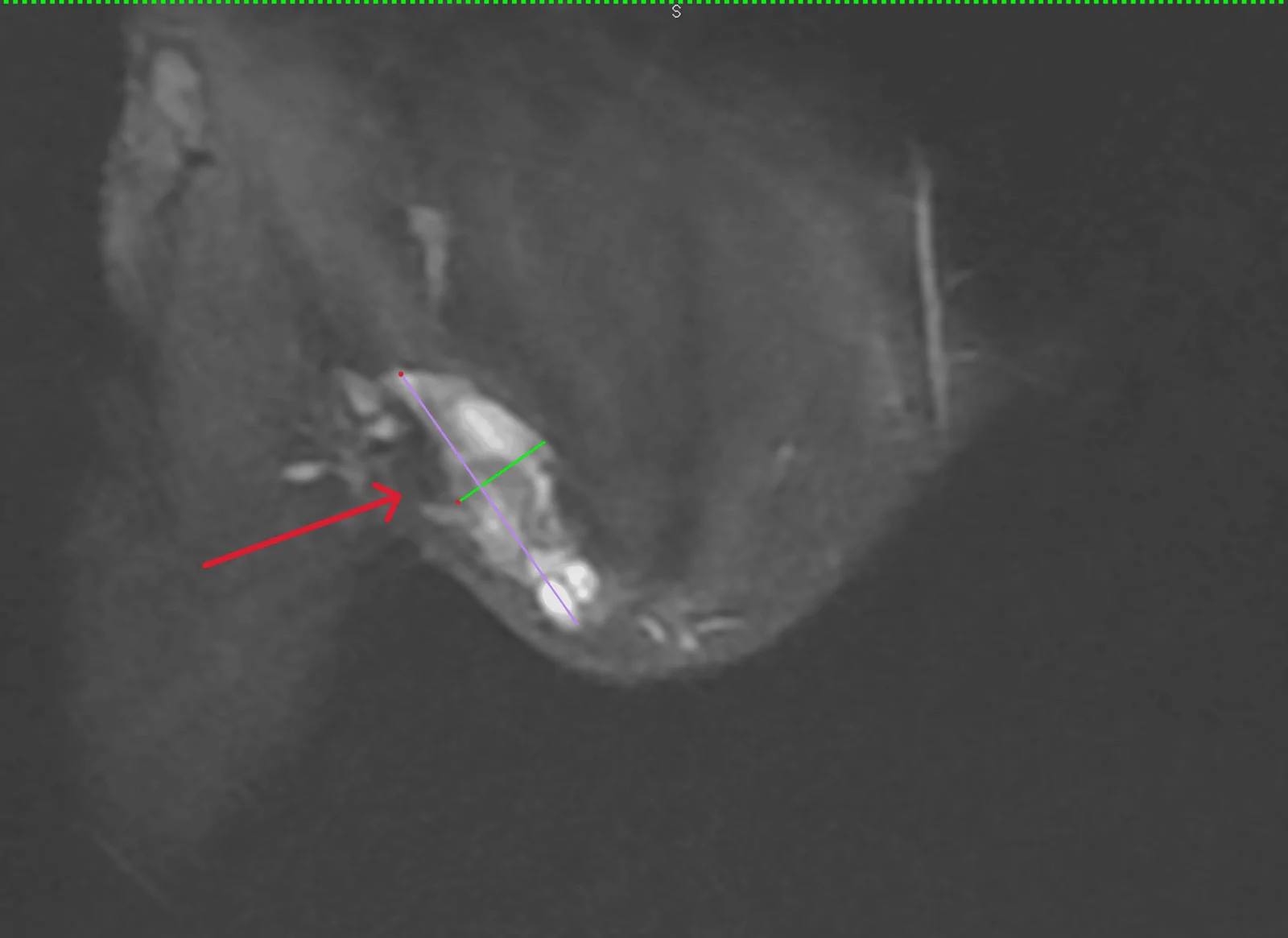
Coronal short tau inversion recovery magnetic resonance imaging of the pelvis and right inguinal region It demonstrates a multiloculated elongated hyperintense cystic lesion (red arrow), measuring approximately 6.3×2.2 cm, extending along the course of the right inguinal canal toward the right labium majus. The lesion follows fluid signal intensity and shows thin internal septation-like components without evidence of surrounding soft tissue invasion.

**Figure 4:**
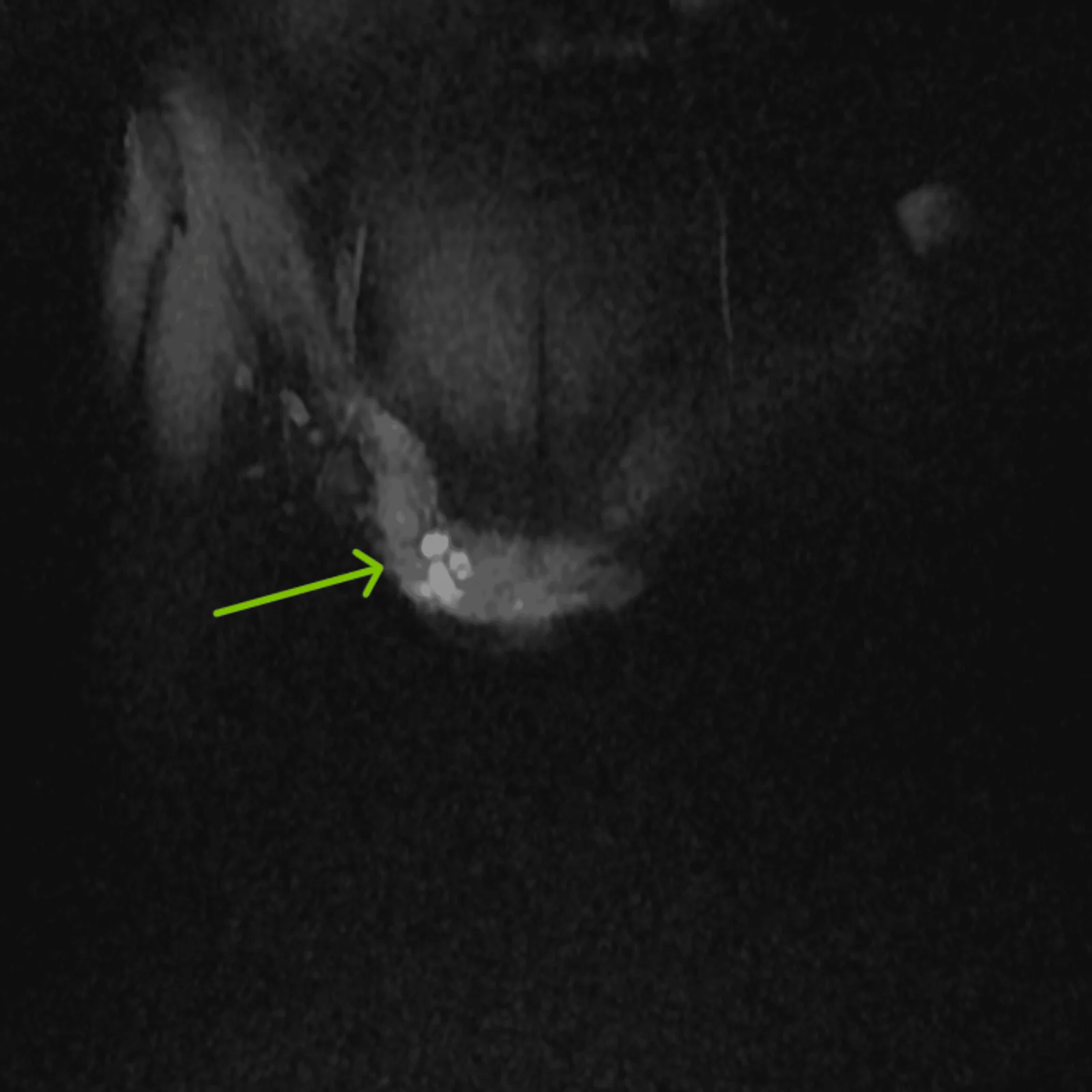
Coronal short tau inversion recovery magnetic resonance imaging showing the inferior extent of the lesion The cystic lesion is seen extending from the region of the deep inguinal ring through the inguinal canal into the right labium majus. Multiple small rounded fluid-filled locules are noted inferiorly (green arrow). No definite communication with the peritoneal cavity is identified on the available images.

**Figure 5:**
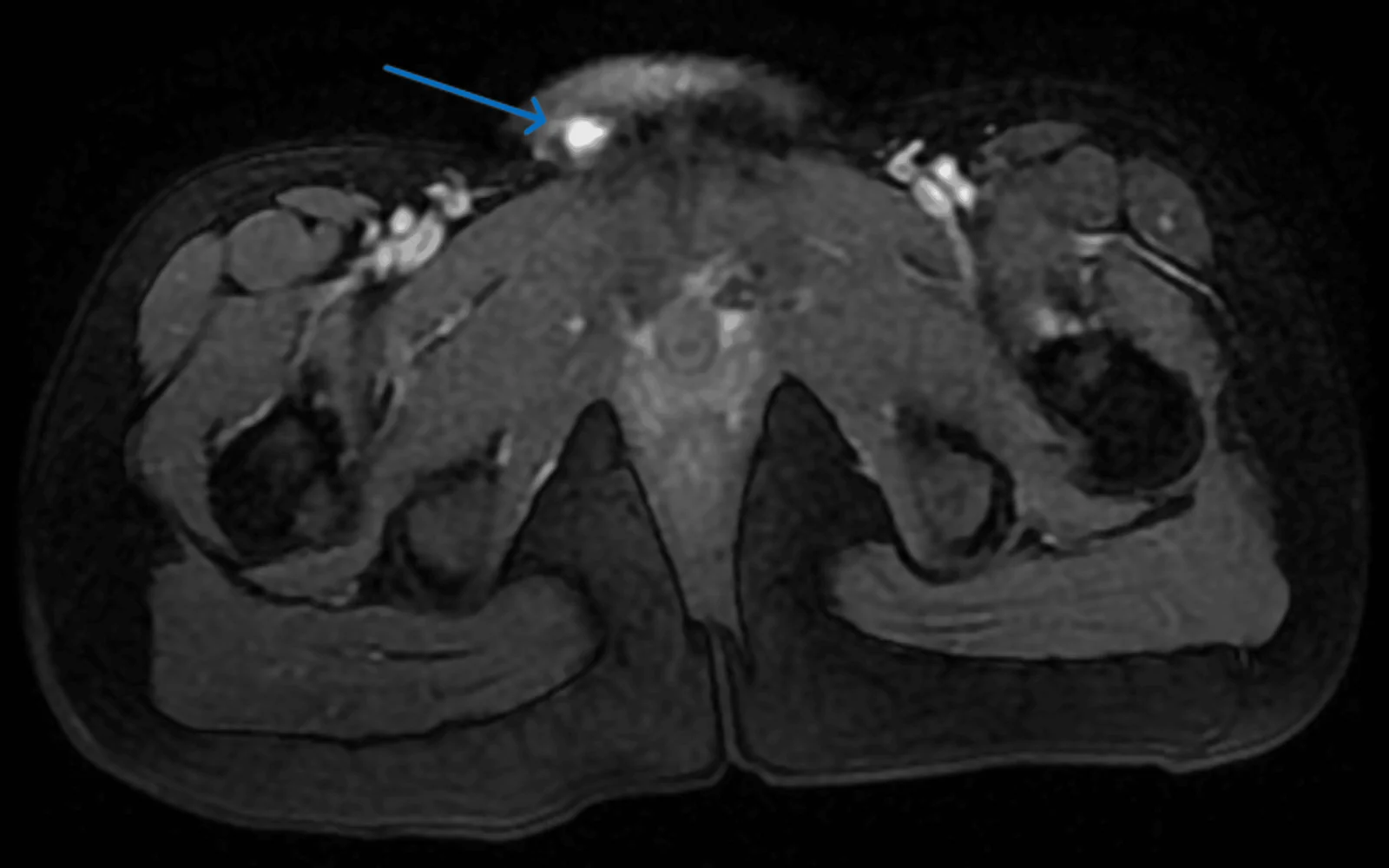
Axial short tau inversion recovery magnetic resonance imaging of the pelvis Axial short tau inversion recovery image demonstrates a well-defined hyperintense cystic lesion within the right inguinal canal (blue arrow). The lesion is located superficial to the pubic region and follows fluid signal intensity. No associated bowel loops, omental fat, or solid components are identified.

Based on the clinical and radiological findings, a diagnosis of hydrocele of the canal of Nuck was made. The patient was referred for surgical consultation; however, surgical intervention had not been performed at the time of manuscript preparation.

## Discussion

Hydrocele of the canal of Nuck is a rare developmental abnormality resulting from failure of obliteration of the processus vaginalis in women. Although well recognized in pediatric populations, it remains an uncommon diagnosis in adult women and is often overlooked clinically because of its nonspecific presentation [3]. Patients typically present with an inguinal or labial swelling that may be painless or associated with intermittent discomfort, frequently leading to an initial diagnosis of inguinal hernia [4].

The differential diagnosis of a female inguinal mass is broad and includes indirect inguinal hernia, lymphadenopathy, Bartholin cyst, abscess, cystic lymphangioma, endometriosis of the round ligament, and soft tissue neoplasms [5]. Consequently, imaging plays a crucial role in establishing the diagnosis and narrowing the differential considerations.

Ultrasonography is considered the first-line imaging modality for the evaluation of inguinal swellings. Typical sonographic findings include a well-defined anechoic or hypoechoic cystic lesion within the inguinal canal, often tubular or sausage-shaped in morphology, with posterior acoustic enhancement and absence of internal vascularity on color Doppler examination [6]. In the present case, ultrasound demonstrated two tubular cystic lesions within the inguinal canal extending toward the right labium majus, without evidence of herniated bowel loops or omental fat, findings strongly suggestive of hydrocele of the canal of Nuck.

MRI serves as an excellent adjunctive modality by providing superior soft tissue characterization and anatomical delineation. Canal of Nuck hydroceles typically appear hypointense on T1-weighted images and hyperintense on T2-weighted sequences owing to their fluid content [7]. Internal septations may occasionally be encountered, particularly in chronic lesions [8]. The MRI findings in our patient, including a tubular cystic lesion extending from the deep inguinal ring through the superficial inguinal ring to the labium majus with T1 hypointensity, T2/STIR hyperintensity, and thin internal septations, were characteristic of this entity.

Hydroceles of the canal of Nuck have traditionally been classified into communicating, non-communicating (encysted), and hourglass types based on their relationship to the peritoneal cavity [9]. The absence of demonstrable communication with the peritoneal cavity in the present case favored the diagnosis of an encysted hydrocele, which is the most frequently reported subtype.

Definitive treatment is surgical excision with closure of any patent processus vaginalis when present [10]. Surgical management not only relieves symptoms but also provides histopathological confirmation and excludes rare mimics. Although our patient has not yet undergone surgery, the characteristic ultrasound and MRI findings allowed a confident radiological diagnosis and facilitated appropriate surgical referral.

This case highlights the importance of considering hydrocele of the canal of Nuck in the differential diagnosis of inguinal swelling in women. Recognition of its characteristic imaging features can improve preoperative diagnostic accuracy and prevent misdiagnosis as a more common inguinal hernia.

## Conclusions

Hydrocele of the canal of Nuck is a rare but important differential diagnosis of inguinal swelling in women and is frequently mistaken for an inguinal hernia on clinical examination. Awareness of this entity and its characteristic imaging features is essential for accurate preoperative diagnosis. Ultrasonography serves as an effective initial diagnostic modality, while MRI provides excellent anatomical delineation and confirms the cystic nature and extent of the lesion. Recognition of these findings can facilitate appropriate surgical referral and help avoid diagnostic delays or unnecessary interventions. A limitation of this case is the absence of surgical and histopathological confirmation; however, the characteristic ultrasound and MRI findings supported the radiological diagnosis.
